# Retraction: LncRNA SRA1 is downregulated in HPV-negative cervical squamous cell carcinoma (CSCC) and regulates cancer cell behaviors

**DOI:** 10.1042/BSR-20191226_RET

**Published:** 2021-02-01

**Keywords:** cervical squamous cell carcinoma, lncRNA SRA1, HPV, miR-9

This article is being retracted from *Bioscience Reports* at the request of the Editorial Board following receipt of a notification from a reader, alerting the Editorial Board to a potential concern with the ethics statement in the article, which had similarities with other published papers and did not match the affiliations of any of the authors.

The authors were contacted by the Journal, but did not respond to any correspondence from the Editorial Office. Subsequently, the Editorial Office has been contacted by the authors to state that they were unaware of the submission to the Journal and that the email addresses used were not theirs (despite this correspondence email being the same as that used throughout the article history and as listed in the Version of Record). In this latest correspondence from the authors, they ask that the paper be withdrawn. The Editor-in-Chief and Editorial Board agree with the retraction.

